# Epidemiology, Risk Factors, and Prevention of Head and Neck Squamous Cell Carcinoma

**DOI:** 10.3390/medsci11020042

**Published:** 2023-06-13

**Authors:** Adam Barsouk, John Sukumar Aluru, Prashanth Rawla, Kalyan Saginala, Alexander Barsouk

**Affiliations:** 1 Internal Medicine, Hospital of the University of Pennsylvania, Philadelphia, PA 19104, USA; adambarsouk@comcast.net; 2 Elucid Bioimaging, Boston, MA 02216, USA; john.aluru@elucid.com; 3 Parrish Medical Center, Titusville, FL 32796, USA; 4 Plains Regional Medical Group Internal Medicine, Clovis, NM 88101, USA; drsaginala@gmail.com; 5 Hematologist-Oncologist, Allegheny Health Network, Pittsburgh, PA 15212, USA; alexbarsouk@comcast.net

**Keywords:** head and neck cancer, epidemiology, incidence, mortality, prevention, risk factors

## Abstract

Head and neck squamous cell carcinoma (HNSCC) is a group of malignancies, involving the oral cavity, pharynx, hypopharynx, larynx, nasal cavity, and salivary glands, that together compose the seventh most common cancer diagnosis worldwide. With 890,000 new cases and 450,000 deaths annually per GLOBOCAN estimates, HNSCC accounts for roughly 4.5% of cancer diagnoses and deaths. In the developing world, the incidence of HNSCC is growing with increasing consumption of tobacco (smoked or chewed), alcohol, and areca nut (betel quid). Alcohol and tobacco have a synergistic effect, with the heavy consumption of both increasing HNSCC risk 40-fold. In developed nations, HPV-related HNSCC surpasses tobacco- and alcohol-related disease. HPV-related HNSCC more commonly affects the oropharynx, hypopharynx, and larynx than the oral cavity, and is associated with a significantly longer median survival (130 months vs. 20 months). Discrepancies in etiology as well as disparities in lifestyle choices and access to healthcare may account for the greater incidence and poorer survival of HNSCC among minority and lower-socioeconomic-status communities in developed nations. Pharmacotherapy and counseling together have been shown to be effective in promoting smoking and alcohol cessation. Education on cancer risk and community engagement have reduced areca nut consumption in Asia as well as in diaspora communities. HPV vaccination, starting at age 11–12 for both sexes, has been shown to reduce the prevalence of high-risk HPV serologies and prevent pre-cancerous lesions of the cervix, vagina, and vulva. As of 2020, 58.6% of eligible adolescents in the US have received the full two-vaccine series. Increased adoption of vaccination, education on safe sex practices, and routine visual oral screening for high-risk patients would curb growing HNSCC incidence in developed nations.

## 1. Introduction

Head and neck squamous cell carcinoma (HNSCC) refers to a group of malignancies that arise from the squamous cells lining the tissues of the head and neck region, including the oral cavity, hypopharynx, nasopharynx, oropharynx, lip, nasal cavity, paranasal sinuses, and salivary glands. These organs function to facilitate respiration and swallowing, as well as filter and humidify the air inspired. HNSCC is a significant health concern worldwide, with its incidence and mortality rates displaying substantial variation across different geographic locations and demographic characteristics. Certain populations are particularly susceptible to HNSCC, with higher rates observed among men, older adults, and individuals of lower socioeconomic status. Understanding these epidemiological patterns is vital for effectively addressing the burden of HNSCC and implementing targeted preventive measures.

In this comprehensive review, we aim to delve into the epidemiology of HNSCC, exploring various aspects such as the incidence and mortality rates associated with this malignancy. By examining the geographical and demographic factors that contribute to HNSCC disparities, we can gain insights into the underlying mechanisms and risk factors involved. Additionally, we will thoroughly examine the most common risk factors associated with HNSCC. These risk factors range from well-established ones such as tobacco smoking and alcohol consumption to emerging factors such as viral infections, including human papillomavirus (HPV) and Epstein–Barr virus (EBV) infections. Understanding the multifaceted nature of these risk factors is crucial for devising effective prevention and intervention strategies that can help reduce the incidence of HNSCC. Furthermore, we will explore evidence-based prevention strategies for HNSCC. This will encompass various approaches, including lifestyle modifications, early detection methods, and vaccination against high-risk viral infections. By highlighting these prevention strategies, we aim to provide healthcare professionals and policymakers with valuable information to guide their efforts in reducing the burden of HNSCC and improving patient outcomes.

Overall, this review seeks to provide a comprehensive overview of the epidemiology of HNSCC, shedding light on its incidence and mortality rates, the most common risk factors, and evidence-based prevention strategies. By consolidating current knowledge in these areas, we hope to contribute to a better understanding of HNSCC and pave the way for more effective prevention and management strategies to combat this challenging disease.

## 2. Epidemiology

### 2.1. Global

**Figure 1:**
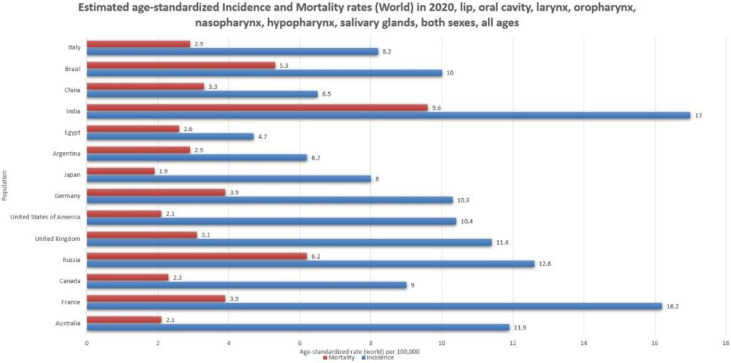
Bar chart showing estimated age-standardized incidence and mortality rates (world) in 2020 for lip, oral cavity, hypopharynx, nasopharynx, oropharynx, and salivary gland cancers, including both sexes and all ages. Data obtained from Globocan 2020 [2].

Per the latest GLOBOCAN estimates (2020), HNSCC is the seventh most common cancer globally, accounting for an estimated 890,000 new cases (roughly 4.5% of all cancer diagnoses around the world) and 450,000 deaths per year (roughly 4.6% of global cancer deaths) [Figure 1]. The incidence includes approximately 380,000 cases of cancer of the lip and oral cavity, 185,000 of the larynx, 133,000 of the nasopharynx, 98,000 of the oropharynx, 84,000 of the hypopharynx, and 54,000 of the salivary glands [1].

The incidence and mortality rates of head and neck squamous cell carcinoma vary widely by geographic region and demographic characteristics. Globally, HNSCC is more common in men than in women, with a male-to-female ratio of approximately 2:1, and in adults over 50 years of age [1]. The incidence rates of HNSCC are highest in South and Southeast Asia (where the chewing of the carcinogenic areca nut is prevalent) [3], followed by Central and Eastern Europe, and South America [1]. The highest incidence rates are observed in India, where tobacco (with or without the areca nut) accounts for up to 80% of all HNSCC cases [4].

**Figure 2:**
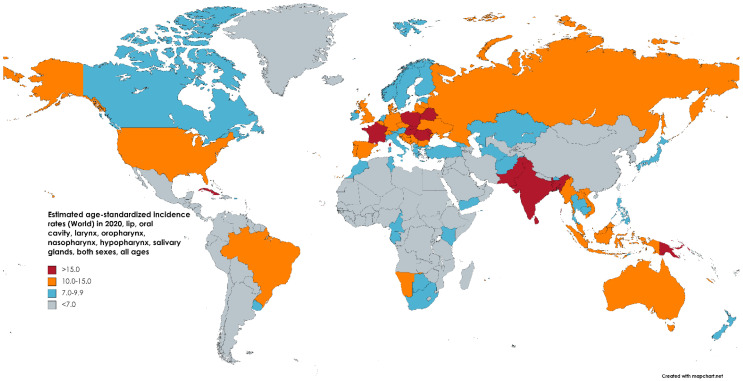
Map showing estimated age-standardized incidence rates (world) in 2020 for lip, oral cavity, hypopharynx, nasopharynx, oropharynx, and salivary gland cancers, including both sexes and all ages. Created with mapchart.net. Data obtained from Globocan 2020 [2].

**Figure 3:**
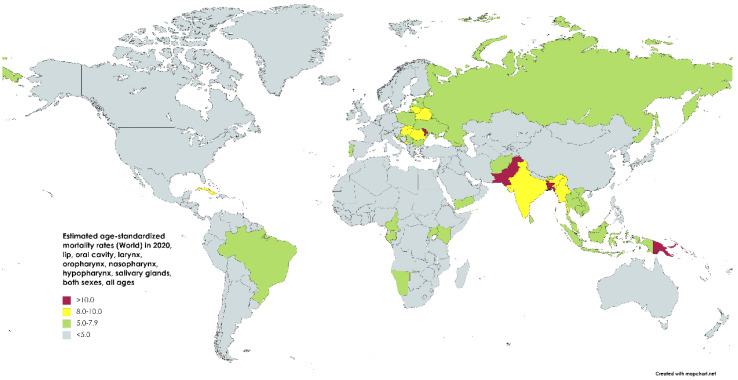
Map showing estimated age-standardized mortality rates (world) in 2020 for lip, oral cavity, hypopharynx, nasopharynx, oropharynx, and salivary gland cancers, including both sexes and all ages. Created with mapchart.net. Data obtained from Globocan 2020 [2].

The global incidence of head and neck squamous cell carcinoma has been increasing in many countries, particularly in younger populations, with a predicted 30% annual increase in incidence by 2030 [Figure 2] [1]. This trend is partly attributed to changes in lifestyle factors, such as increased alcohol consumption and tobacco use in developing nations, as well as the growing prevalence of human papillomavirus (HPV)-related oropharyngeal cancer. It is estimated that HPV will overtake tobacco as the leading contributor to the global HNSCC cancer burden, causing the incidence of oropharyngeal HNSCC to surpass that of oral cancer (which is predominantly tobacco-related) [1,5]. Similarly, over the past decade, laryngeal cancer cases have increased by 23% [5]. Younger women in developed nations have seen a marked growth in incidence, likely due to changes in sex-specific cultural expectations for tobacco and alcohol consumption as well as a growing HPV burden [6,7]. In Japan, the incidence of oropharyngeal, oral cavity, and salivary gland tumors has gone up, more for women than men, while nasopharyngeal and laryngeal tumor incidence has gone down (which are more associated with the Epstein–Barr virus in East Asian populations than with HPV) [8]. Likewise, in the UK, oropharyngeal HNSCC has increased by 7.3% among men and 6.5% among women, and oral cancer has increased by 2.8% and 3.0% for men and women, respectively, with the greatest growth seen in those of lower socioeconomic status [Figure 3] [9,10].

### 2.2. United States

In the United States, per the SEER estimates, around 54,000 new cases of head and neck squamous cell carcinoma were diagnosed in 2022 (roughly 3% of all malignancies), with an estimated 11,230 deaths, accounting for roughly 2% of all cancer deaths. Some 27% of cases are localized at diagnosis (stages I and II), 51% are locally advanced (stages III-IVB), and 15% are distantly metastatic (stage IVC). The 5-year survival is 68.5%, but it varies by stage at diagnosis. The 5-year survival is 86.6% for localized disease, 69.1% for locally advanced disease, and 39.3% for metastatic disease. The median age at diagnosis is 64, with around half of patients diagnosed between the ages of 55 and 74. The incidence among all males is 17.2/100,000 but is greatest for non-Hispanic White (20.1/100,000) and American Indian and Alaska Native (17.5/100,000) patients, with the lowest incidence in Hispanic (10.3/100,000) and Asian and Pacific Islander (12.0/100,000) patients. The incidence among females is nearly three times lower at 6.4/100,000 and is similarly highest for non-Hispanic White (7.1/100,000) and Asian and Pacific Islander (5.0/100,000) patients, with the lowest incidence in Hispanic (4.3/100,000) and Black (5.0/100,000) patients. The incidence rates of HNSCC in the United States have declined overall by approximately 14% since 1975, largely due to decreases in tobacco use. However, since the nadir in 2003, incidence rates have increased by 15.5% [11]. This growth has coincided with the replacement of tobacco-related HNSCC with HPV-related disease, with oropharyngeal disease set to surpass oral disease [12].

Since 1975, head and neck squamous cell carcinoma mortality in the US has fallen by 44%, down to a mortality rate of 2.5/100,000 persons in 2020. This has been accompanied by an increase in 5-year survival from 54.6% in 1975 to 68% in 2018. Survival rates remain disparate based on the stage of diagnosis. The 5-year survival for localized disease is 86.3%, decreasing to 69.0% for locally advanced and 40.4% for metastatic disease [11,12]. The increase in survival among developed nations has been multifactorial, including the shift to HPV-related cases (which portend better prognosis), earlier detection due to screening, advances in robotic surgical resection and stereotactic radiation, as well as the development of immunotherapies (checkpoint inhibitors) for neoadjuvant [13], adjuvant [14], and metastatic systemic therapy [15,16]. Access to screening and advanced therapies remains a contributor to disparate survival statistics, with racial minorities and those living in low-socioeconomic-status urban and rural communities (e.g., Appalachia) suffering greater incidence and lower overall survival [17,18]. An analysis of HNSCC diagnoses at one academic institution during the COVID-19 pandemic suggested a larger proportion of late-stage diagnoses and worsened tumor burden following quarantine, potentially exacerbating disparities in survival [19].

## 3. Risk Factors

The primary risk factors commonly linked to head and neck cancer encompass tobacco, alcohol consumption, using areca nut, human papillomavirus (HPV) infection (particularly for oropharyngeal cancers), and Epstein–Barr virus (EBV) infection (especially prevalent in Asia, particularly for nasopharyngeal cancers).

### 3.1. Tobacco

Tobacco use remains the leading risk factor for head and neck squamous cell carcinoma, accounting for an estimated 75% of all cases according to a study of cases in Western Europe [20]. A national survey from the UK implicated tobacco in 70% of oral and pharyngeal HNSCC cases [21], while data from East Asia have implicated tobacco in 2.8–25% of cases. Tobacco use is increasing across developing nations as economic progress increases household disposable income. In developed nations, tobacco use has declined overall but increased in certain demographics, commonly women [22,23]. Tobacco contains numerous carcinogenic chemicals such as polycyclic aromatic hydrocarbons, nitrosamines, aromatic amines, and aldehydes which are released during high-temperature combustion that are known to damage DNA in the cells of the oropharynx and lead to the development of cancer. Heavy cigarette smokers have a 5–25-fold increased risk of HNSCC as compared to non-smokers [24], while pipe and cigar smokers are at a lesser, yet still increased, risk [25]. Regular chewing tobacco use is also associated with a 1.7 odds ratio (OR) for HNSCC and 3.0 OR for oral cancer specifically [26]. Individuals with certain genetic and metabolic predispositions, including concurrent heavy drinking, are at the highest risk of developing HNSCC with smoking [25]. Second-hand smoke exposure in childhood was found to have a 1.28 OR for HNSCC, adjusted for smoking, drinking, and HPV status [27].

### 3.2. Alcohol

Alcohol consumption among nonsmokers is estimated to account for 4% of head and neck squamous cell carcinoma cases globally [28]. The risk of HNSCC increases in a dose-dependent manner with the amount and frequency of alcohol consumption (relative risk 1.3× for all drinkers and 2.5× for heavy (>50 g/day) drinkers), with the highest risk observed among individuals who consume more than three alcoholic drinks per day [28]. The risk of HNC also varies by the type of alcohol consumed, with higher risks observed among individuals who consume spirits (e.g., whiskey or vodka) than among those who consume beer or wine, although this effect may be nonsignificant when adjusted for alcohol concentration [29,30,31]. Alcohol’s property as a solvent increases the mucosal tissue’s susceptibility to carcinogens such as smoke or nitrites in food. Acetaldehyde, produced from ethanol by alcohol dehydrogenase, has also been shown to be mutagenic. Acetaldehyde is responsible for many of the symptoms of heavy alcohol consumption, such as headaches and flushing, and its conversion is blocked by disulfiram and other drugs with similar properties, such as metronidazole or abacavir, resulting in reactions with drinking [31,32]. Interestingly, patients with variants in acetaldehyde dehydrogenase, the enzyme that breaks down acetaldehyde, have been found to be associated with greater HNSCC risk with heavy alcohol consumption, greater risk of resistance to chemoradiotherapy, and overall poor prognosis. Variants in acetaldehyde dehydrogenase are also responsible for the phenomenon of flushing with alcohol consumption, common in East Asian populations [32].

Alcohol and tobacco have a multiplicative effect on head and neck squamous cell carcinoma. Increases in alcohol and tobacco consumption are projected to be the major contributors to growing global HNSCC incidence over the coming decades, particularly in developing nations. Studies from the UK [21], China [22], and Lebanon [33] all implicate alcohol consumption in 32–37% of oropharyngeal HNSCC diagnoses, with approximately 90% of these cases also reporting a smoking history [28,34]. Worldwide, tobacco or alcohol account for 72% (95% CI 61–79%) of HNSCC cases, with 35% (nearly half) attributed to both combined [28]. In fact, concurrent heavy alcohol and tobacco use has been shown to increase HNSCC risk 40-fold [31]. Interestingly, among Black Americans, the association between cigarette smoking and HNSCC was greater than for White Americans, but the alcohol consumption risk was unchanged between the groups, suggesting differences in tobacco product quality or metabolism by race [35].

### 3.3. Areca Nut (Betel Quid)

In South and Southeast Asia and Polynesia, chewing of the areca nut, also known as betel quid, accounts for over half of oral and oropharyngeal head and neck squamous cell carcinoma cases [36]. The prevalence of consumption ranges from 33.8% in Sri Lanka [37] to 76.8% in the Solomon Islands [38]. Chewing can be part of rituals or recreational, as the compound has psychoactive effects via antagonism of GABA receptors, resulting in alertness, euphoria, and appetite suppression [39]. In fact, around 600 million people chew areca nuts worldwide, and it is considered the fifth most commonly used psychoactive agent after alcohol, nicotine, caffeine, and cannabis. In some of these nations, Areca nut is the most affordable and accessible stimulant and appetite suppressant, thus making its usage particularly prevalent in rural and underprivileged populations [36,39]. Areca nut can often be prepared with tobacco added, which may account for disparate risk calculations. A study from India found a 3× increased risk of HNSCC with areca chewing without tobacco and an 8× risk with tobacco, while a study from Taiwan reported a 10× risk without tobacco. Both studies reported positive dose–response curves [36].

### 3.4. Human Papillomavirus (HPV) Infection

HPV accounts for 72% of all head and neck squamous cell carcinoma cases in developed nations, as compared to 13% of cases in developing nations (as indicated by p16 positivity on immunohistochemistry) [40]. Over 20 high-risk HYPV serotypes have been identified, distinguished by the protein capsid. HPV16 is responsible for 85–90% of HPV-related oropharyngeal HNSCC cases in North America, in contrast to cervical cancer where HPV16 and 18 both account for 50–75% of cases [41]. Oncogenesis occurs with viral infection of basal keratinocytes, exposed to micro-abrasions caused by sexual contact [42]. The risk factors for HPV-associated oropharyngeal cancer include a history of multiple sexual partners, anal sex, oral sex (with the mouth on female genitalia conferring the highest risk), and a weakened immune system. Comorbidity with human immunodeficiency virus (HIV) is common, with women with HIV having a 1.5–2.5 times greater risk of HPV. Globally, those with >4 oral sex partners have an OR of 2.25 (95% CI 1.42–3.58) of HNSCC [43]. The prevalence of HPV-associated oropharyngeal cancer has increased in developing nations in recent years (225% in the US from 1984 to 2004), particularly among young adults [44]. In the US, HNSCC has surpassed cervical cancer as the leading HPV-associated cancer [45]. Among US demographics, White men <45 years saw the greatest growth in incidence, with a 5.1% annual increase from 2008 to 2012. By contrast, the incidence of non-HPV-associated HNSCC is roughly 50% higher among Black Americans [46]. Likewise, in the UK, cases of HPV-related HNSCC rose by 51% from 1989 to 2006 [47]. HPV-positive HNSCCs are associated with greater infiltration of B-cells into the tumor microenvironment, have fewer genetic mutations, and have an intact apoptotic response, which may explain the improved prognosis and superior response to radio- and immunotherapy [42,48]. HPV positivity portends a significantly longer median survival among HNSCC patients (130 vs. 20 months) [49], independently reducing the risk of death by 64% when adjusted for risk factors [50]. HPV-positive HNSCC is now staged differently due to the large disparity in survival [51].

### 3.5. Opium

The use of opium has been linked to an elevated risk of laryngeal cancer. Opium, an illicit substance derived from the poppy plant, is obtained from the juice of the unripe seedpod and contains several alkaloids. According to the International Agency for Research on Cancer, opium is classified as carcinogenic to humans when smoked or consumed in various forms, including raw, dross, or sap opium. For instance, findings from the Golestan Cohort Study (GCS), which examined 50,045 patients in Iran, demonstrated that opium use was associated with an increased risk of developing laryngeal cancer that correlated with the dosage [52].

### 3.6. Other Viral Infections

Nasopharyngeal carcinoma, while uncommon in most populations, is prevalent in southern China, ranking among the most frequently diagnosed cancers in the region. Research has established the significant involvement of the Epstein–Barr virus (EBV) as the primary causative factor in the development of nasopharyngeal carcinoma. Additionally, chronic hepatitis C virus (HCV) infection has a robust association with the occurrence of hepatocellular carcinoma, and it has also been linked to various other malignancies such as lymphoproliferative disorders.

### 3.7. Immunodeficiency

Immunodeficiency resulting from HIV infection or solid organ transplantation has been linked to a heightened susceptibility to cancer in the head and neck area. HIV-infected individuals exhibit a higher occurrence of various non-AIDS-defining malignancies. Notably, there is a two- to threefold rise in the incidence of HNSCC among HIV-infected patients. Similarly, individuals who have undergone solid organ transplantation face an elevated risk of developing cancer, including malignancies originating in the head and neck. Moreover, patients who have received a bone marrow transplant, despite lacking conventional risk factors, are at an increased risk of head and neck cancer, particularly in the oral cavity [53].

### 3.8. Other Risk Factors

Other risk factors for head and neck squamous cell carcinoma include exposure to occupational or environmental carcinogens (e.g., asbestos or wood dust), poor oral hygiene, poor nutrition, and genetic predisposition. Chronic inflammation and infection of the oral cavity, such as chronic periodontitis, have also been linked to an increased risk of HNC [6,7,16,54]. Individuals diagnosed with Fanconi anemia face a substantial predisposition to various malignancies, such as HNSCC, myelodysplastic syndrome, and acute myelocytic leukemia.

Certain dietary factors can play a role in either protecting individuals from head and neck cancer or heightening their vulnerability to specific diseases. Numerous studies have demonstrated a protective effect linked to higher intake of fruits and vegetables. Conversely, case-control studies indicate that frequent consumption of preserved meats, which contain elevated levels of added nitrites, may increase the risk of nasopharyngeal carcinoma.

The administration of prior radiation therapy, whether for malignant or benign conditions, has been associated with an increased incidence of thyroid cancer, salivary gland tumors, squamous cell carcinomas (SCCs), and sarcomas. While this connection is indeed present, it should be noted that there is a considerable time delay before any potential adverse effects manifest, and the overall risk remains relatively low.

## 4. Prevention

The prevention of head and neck squamous cell carcinoma involves public health initiatives aimed at increasing oral screening and HPV vaccination, reducing exposure to tobacco, alcohol, and areca nut, and promoting a healthy lifestyle. The promotion of healthy diets, such as those high in fruits and vegetables, and good oral hygiene can also reduce the risk of HNSCC.

### 4.1. Smoking Cessation

Counseling by healthcare practitioners and pharmacotherapy (nicotine replacement, varenicline, and/or bupropion) for smoking cessation in tangent have been shown to be most effective in promoting smoking cessation among head and neck squamous cell carcinoma patients in large systematic reviews. The average smoker requires over 10 attempts to discontinue, though this figure can be dramatically reduced by a combination of pharmaco- and psychotherapy [53,54]. In the general population, maintained abstinence remains low (18–30%) even with varenicline therapy, highlighting the need for research into alternative mechanisms of targeting cravings. Other medications, including GLP-1 agonists such as semaglutide and naltrexone, are under investigation for tobacco addiction [55].

### 4.2. Decreasing Alcohol Consumption

The World Health Organization (WHO) recommends a maximum daily intake of 30 g of alcohol for men and 20 g for women. Pharmacotherapy for alcohol use disorder (approved agents include naltrexone, acamprosate, nalmefene, and disulfiram) was found, in one US study, to be prescribed to only 3.6% of eligible patients [56]. Pharmacotherapy plus cognitive behavioral therapy, or other forms of evidence-based counseling, have been shown to improve cessation rates over pharmacotherapy alone [57]. Patients undergoing alcohol withdrawal are recommended for inpatient admission due to the risk of seizure and delirium tremens. Communities of lower socioeconomic status, such as Black Americans, are at higher risk of concurrent alcohol and tobacco consumption and should be targeted in public health initiatives to increase participation in regular wellness checkups and screenings, and substance use programs. Public health initiatives engaging community leaders such as pastors and barbers have proven especially effective in health promotion in US Black communities [58].

### 4.3. Ceasing Areca Nut Consumption

Studies from Taiwan, India, and China suggest that ceasing areca nut consumption would prevent roughly half of oral cancers in those nations [36]. In northern Thailand, head and neck squamous cell carcinoma incidence dropped 2–3-fold from 1988 to 1999 due to concerted efforts to discourage Areca nut consumption, particularly among young adults [59]. A study from Guam found that while the average tobacco smoker required 11.5 attempts before successfully quitting, that figure was only 5.2 for habitual areca users [60]. Programs aimed at increasing awareness of areca nut carcinogenicity have been successful in promoting cessation. The most successful interventions will involve engaging community leaders, particularly in refugee communities with lower medical literacy such as the Hmong and Karen [61,62].

### 4.4. HPV Screening and Vaccination

HPV vaccination is currently approved as a two-dose routine vaccine beginning at ages 11–12, though it may be administered through to the age of 26 according to the US Centers for Disease Control. As HPV vaccination was first approved in 2006 for adolescent females, the latency period has not yet been adequate to establish the impact of vaccination programs on the incidence of HNSCC [63]. Vaccination has been shown, in a US cross-sectional study, to reduce the prevalence of oral HPV 6, 11, 16, and 18 (the latter two being high-risk serotypes) by 88.2%. Following the implementation of the vaccine, the US Centers for Disease Control found a 64% decrease in high-risk HPV among sexually active women under 20, and a 34% decrease in women aged 20–24 [64]. Randomized trials for the Cervarix and Gardasil vaccines have also shown a significant reduction in cervical, vaginal, and vulvar HPV infection and HPV-associated precancerous lesions [65]. The quadrivalent vaccines were approved for adolescent and young adult men in 2009, and the most recent vaccines now cover nine HPV serotypes. The rate of adolescents who received the full two doses of the HPV vaccine increased from 54.2% in 2019 to 58.6% in 2020 [66], though the proportion may have since declined as the COVID-19 quarantine disrupted annual wellness exams.

Genital HPV screening for cervical cancer consists of regular pap smears. The pap smear aims to sample cells from the cervix to look under the microscope for dysplastic changes, i.e., changes in the cell structure that suggest it has started on its path to becoming cancerous. This screening is recommended by the USPSTF for anyone with a cervix every three years beginning at age 21 until age 65. Microscopy can also be combined with HPV molecular testing via PCR. This combined approach can be started at 30 and only needs to be done every five years instead of every three. The ACS recommends HPV testing every five years beginning at 25 without the need for cervical sampling. HPV testing is not recommended before age 25–30, as many women may have the infection but their immune system is robust enough to clear it, and thus the risk of cancer being caused by the HPV is much lower. Pap screenings have reduced US cervical cancer death rates by over 70% since the 1960s. Unfortunately, studies around the US found a significant reduction in screening rates during COVID-19, up to 80%, affecting Asian Americans and Pacific Islanders most severely. Patients found to have active HPV infection on a pap smear, or anal swab, can minimize transmission during oral sex, and thus minimize HNSCC risk, by the use of safe sex practices, such as the use of condoms and dental dams. The prevention of HPV-related HNSCC involves promoting awareness of sexual transmission and safe sex practices and increasing HPV vaccination rates.

### 4.5. Secondary Prevention

As of 2017, US dentists are required to perform oral examinations as part of wellness encounters, though national uptake of the practice has been mixed. Data from 1998 found only 15–19% of adults aged >40 reported having had an oral cancer examination [67]. The US Preventative Screening Task Force has found insufficient evidence to recommend regular oral cancer screening for normal-risk adults, although the American Cancer Society does recommend screening [68].

However, among adults with risk factors for head and neck squamous cell carcinoma, there is data to support annual screening. The greatest risk factor for HNSCC is a prior history, which increases the annual risk of a second primary tumor by 2–7%. Patients with premalignant oral leukoplakia have a roughly 12% lifetime risk of oral cancer [69]. Patients with hereditary cancer syndromes such as Fanconi anemia, and Li–Fraumeni and Plummer–Vinson syndromes, as well as immunosuppression, also associated with increased HNSCC risk, should undergo screening. A study of oral screening interventions from India found a greater detection of early-stage cases but did not find a statistically significant mortality rate reduction (0.8, 95% CI 0.5–1.2); however, for males with tobacco and alcohol use, the reduction was significant (0.6, 95% CI 0.4–0.9) [70]. Compared to visual examination, alternative methods such as toluidine blue staining, brush biopsy, or fluorescence imaging have not been found to reduce HNSCC mortality. Routine screening for HPV infection is not currently recommended [71].

## 5. Conclusions

Head and neck squamous cell carcinoma (HNSCC) represents the seventh most commonly diagnosed tumor type, and its incidence and mortality rates are on the rise. This alarming trend can largely be attributed to preventable risk factors such as the ingestion of tobacco, alcohol, areca nut, and sexually-transmitted HPV infections. Notably, in developed nations, HPV-related HNSCC is expected to surpass tobacco- and alcohol-related disease in terms of prevalence. However, advancements in therapy have played a pivotal role in enhancing patient survival rates.

To address the escalating burden of HNSCC, it is imperative to implement comprehensive public health interventions. These interventions should predominantly focus on educating individuals about the risks associated with consuming carcinogenic agents, as well as promoting and facilitating cessation of tobacco, alcohol, and areca nut use. Furthermore, the wider implementation of HPV vaccination and safe sex practices is crucial in preventing HPV-related HNSCC. Lastly, identifying and screening high-risk patients can substantially contribute to early detection and timely intervention.

By employing these multifaceted approaches, it is possible to effectively curb the projected growth in HNSCC cases. A concerted effort by healthcare professionals, policymakers, and the community at large is essential to raise awareness, change behaviors, and implement preventive measures. With a collective commitment to reducing the impact of HNSCC, we can aspire toward a future with improved outcomes and a reduced burden of this devastating disease.

## Data Availability

Not applicable.
